# iCanPlot: Visual Exploration of High-Throughput Omics Data Using Interactive Canvas Plotting

**DOI:** 10.1371/journal.pone.0031690

**Published:** 2012-02-29

**Authors:** Amit U. Sinha, Scott A. Armstrong

**Affiliations:** 1 Division of Hematology/Oncology, Department of Pediatric Oncology, Dana-Farber Cancer Institute, Children's Hospital Boston and Harvard Medical School, Boston, Massachusetts, United States of America; 2 Harvard Stem Cell Institute, Boston, Massachusetts, United States of America; University of Toronto, Canada

## Abstract

Increasing use of high throughput genomic scale assays requires effective visualization and analysis techniques to facilitate data interpretation. Moreover, existing tools often require programming skills, which discourages bench scientists from examining their own data. We have created iCanPlot, a compelling platform for visual data exploration based on the latest technologies. Using the recently adopted HTML5 Canvas element, we have developed a highly interactive tool to visualize tabular data and identify interesting patterns in an intuitive fashion without the need of any specialized computing skills. A module for geneset overlap analysis has been implemented on the Google App Engine platform: when the user selects a region of interest in the plot, the genes in the region are analyzed on the fly. The visualization and analysis are amalgamated for a seamless experience. Further, users can easily upload their data for analysis—which also makes it simple to share the analysis with collaborators. We illustrate the power of iCanPlot by showing an example of how it can be used to interpret histone modifications in the context of gene expression.

## Introduction

With the growing use of high throughput assays, there has been an increase in the complexity of both the “omics” data and computational techniques for visual exploration. Current approaches for visualization include specialized plotting programs (e.g., gnuplot), software packages (e.g., Matlab, R) and plotting libraries specific to a programming language (e.g., matplotlib library for python language). These techniques require specialized computational skills that discourage the investigation of data by experimentalists who generate the data. More importantly, in such approaches, the visualization and analysis are not integrated thus making it difficult for the user to interactively examine the data.

There is a need for applications with intuitive analysis and interactive visualization to provide a low barrier to entry for non-programmers. Innovations in web-based applications have the potential to meet several of these challenges. Web applications such as Galaxy [1], GenePattern [2], Cinteny [3], etc., allow users to upload their data and analyze it online. Though desktop applications are more responsive than web applications, the gap has shrunk with the development of client side web technologies. Such technologies that could be used for visualization include Scalable Vector Graphics (SVG), Flash, HTML5 Canvas, etc. As each of them run in the users' browser (client-side), there is no lag due to communication between the client and server making them suitable for interactive applications. Several SVG based plotting libraries, such as Protovis [4], are available. However, SVG does not scale well for large data sets, as it requires a Document Object Model (DOM) object for each graphic element. Canvas is a new element introduced as part of the core HTML5 standard that allows for dynamic rendering of shapes and images. It also scales well for large genomic data sets with tens of thousands of records.

Web-based software also helps in the physical aspects of using software tools. Unlike the desktop counterparts, applications deployed in the cloud allows the user to focus on using the tool and avoid dealing with issues such as hardware requirement, operating system upgrades, scalability, etc [5]. One such mature platform for serving applications over the web is the Google App Engine (GAE), which provides platform-as-a-service. In this system, the developer deploys his code while the OS, web server, database and other system level software are preconfigured. The users may access the application from any computer or mobile device with a modern browser and an Internet connection.

To demonstrate the effectiveness of an integrated platform, we have developed iCanPlot—a tool for data exploration with a seamless amalgamation of visualization (using HTML5 Canvas element) and analysis (using Google App Engine). Users may upload their data to probe it in a user-friendly manner.

## Results

We present an example to illustrate how iCanPlot can be used effectively and effortlessly to extract biologically meaningful patterns from the data. Say the user's goal is to understand the relationship of histone modifications H3K27me3 and H3K79me2 with each other and with gene expression [6]. The user should process the raw data beforehand to create a table where the rows correspond to genes and columns correspond to gene expression, H3K27me3 and H3K79me2 levels. This processing of data is not coupled with iCanPlot and can be accomplished using user's preferred pipeline. For example, gene expression values could be obtained using RMA summarization and histone methylation levels can be quantified by counting the number of reads in a gene's promoter. Using these values, the users may create a tab-delimited text file, in a simple format documented on the website. Once the file is uploaded to the website, the user is provided a unique URL that the user or his/her collaborators could use for exploring the data.

**Figure 1:**
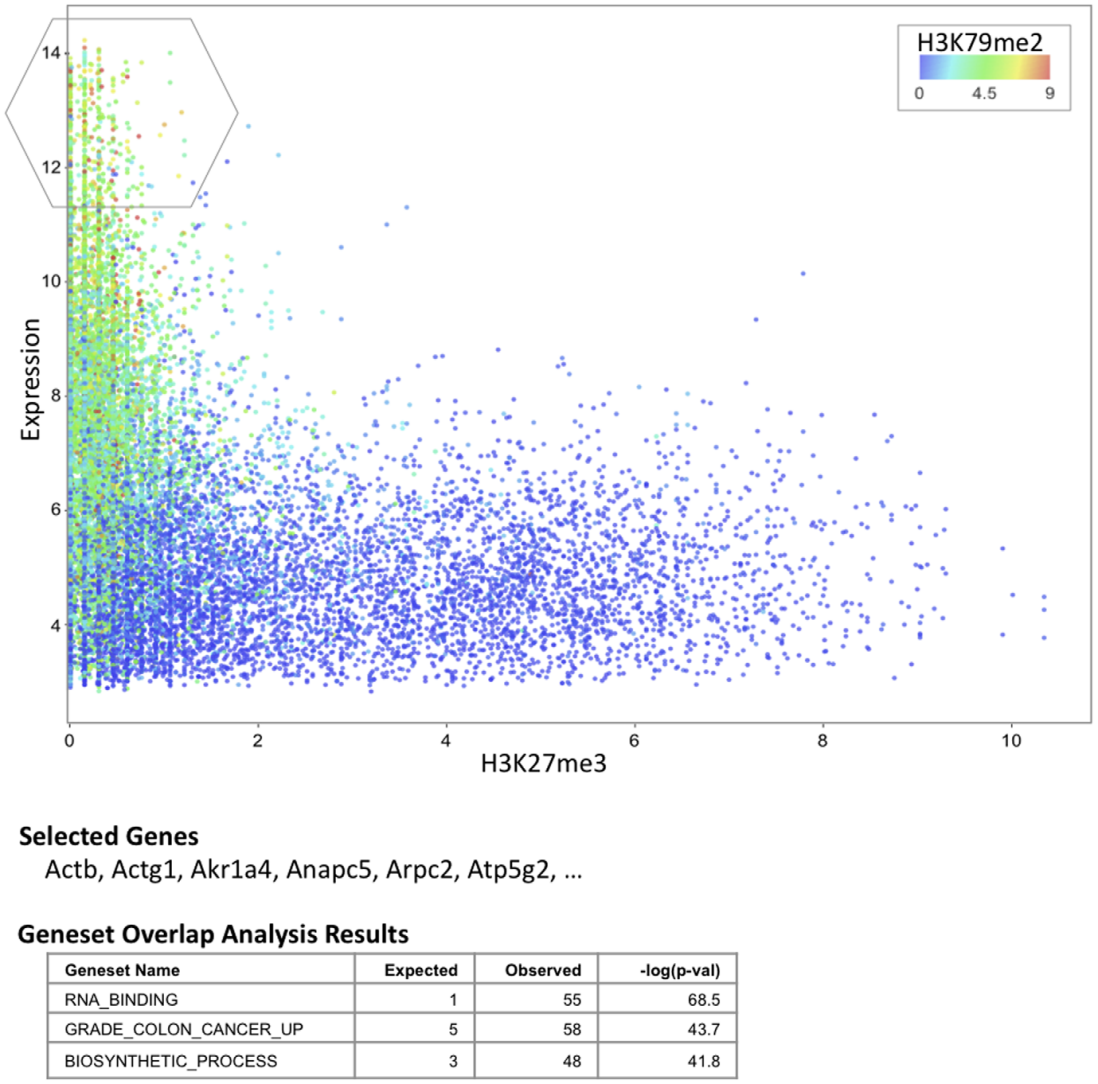
Interactive analysis of histone modification and gene expression in hematopoietic stem cells. Each point represents a gene with the H3K27me3 methylation level on the x-axis and gene expression value on the y-axis. The points are colored by the level of H3K79me2 methylation; genes with red indicating highest and blue lowest methylation levels. The first few genes identified by the user by selecting the region outlined in grey are shown below the plot. Part of the geneset overlap analysis for the selected genes is shown at the bottom.

When the user visits the URL, the data uploaded by the user is retrieved. The columns (in this case gene expression, H3K27me3 and H3K79me2) are made available as possible options for setting the x-axis, y-axis, color and size of the points in the plot. To visualize the relationship between H3K27me3 and gene expression, the user may chose the histone mark H3K27me3 on the x-axis and gene expression on the y-axis. This produces an L-shaped plot that shows that genes either have a) high expression and low H3K27me3 or b) low expression and high H3K27me3 or c) low expression and low H3K27me3. This plot thus presents a convincing visual evidence of the repressive effect of H3K27me3 on gene expression. Further, the user may color the plot by H3K79me2 levels by choosing the option in the color dropdown box. The plot is redrawn in a couple of seconds such that genes with the highest and lowest levels of H3K79me2 are respectively red and blue in color. The user may readily observe that genes with high H3K27me3 are blue while the genes with high expression and low H3K27me3 are green or red. Such a plot indicates that H3K79me2 is associated with high expression, and genes with H3K27me3 have no H3K79me2. Finally, to understand more about the function of the highly expressed genes, a user may draw a region of interest to see the name of genes in the region. This action automatically triggers a geneset overlap analysis and the pathways associated with the selected genes are presented to the user. The final figure and analysis results are shown in figure 1. Thus, using iCanPlot, a user identifies patterns to suggest the repressive role of H3K27me3, the activating role of H3K79me2 and the pathways enriched in highly expressed genes, all within a few seconds.

## Discussion

The current and future generation of genomic data requires state-of-the-art software and hardware for effective visualization and analysis. We present iCanPlot, an interactive tool based on HTML5 Canvas to create responsive and scalable visualization of genomic data. The tool has an intuitive interface that will allow experimentalists to explore their data with ease. An enrichment analysis module was created using the Google App Engine where genes selected by the user are analyzed on the fly. Other types of plot and analysis modules will be added in the future. The underlying plotting library has been designed for reuse that encourages developers to integrate it in their own web applications.

We exemplified the power of iCanPlot by showing how it can be used to interpret histone modification in the context of the gene expression. While other software could do such analysis, using this tool makes the process very simple, user friendly and quick. By integrating visualization and analysis in an interactive fashion, we have created a compelling platform for visual exploration of data that would be of interest to experimentalists because of its ease of use.

## Design and Implementation

### Dynamic Plotting

We have developed a plotting library based on Canvas element to embed a dynamic scatter plot in an HTML page. A typical data set used with iCanPlot consists of a matrix where the rows represent genes and columns represent measurements, e.g., gene expression, histone methylation level, etc. The tool dynamically creates a dropdown menu to choose any combination of measurements for display as x-axis, y-axis, color and size of the plot. This allows the users to interactively plot the measurements against each other to identify biologically interesting patterns. An advantage of using the Canvas element is that it scales very well; a genomic data set with 25,000 points is plotted within 1–2 seconds.

### User Interaction

Users may interact with the plot by selecting the data points of interest by defining an arbitrary selection polygon. To start a selection, a user needs to mark the first node of the polygon by clicking on the plot. Subsequent nodes of the polygon are determined by a click of the mouse on the plot. Finally, double clicking marks the final point of the polygon. Figure 1 shows a region selected by the user and name of the genes inside the polygon are displayed below the plot. Developers may hook analysis modules that are triggered automatically once the user completes the selection.

### Analysis

We have implemented geneset overlap analysis on GAE such that once the users marks a region of interest, the genes in the region are overlapped with genesets from MSigDB [7], which includes Gene Ontology [8] and KEGG [9] genesets, among others. The significant overlaps are presented to the user in a tabular form below the plot. In figure 1, the list at the bottom shows a few of the enriched genesets, expected size of overlap, observed size of the overlap and −log_10_(P-value). These results are generated on the fly and users may interactively analyze the data. Developers who integrate the iCanPlot library into their web application can configure other types of analyses to be triggered with the selected genes.

### User Data Upload

The iCanPlot website allows users to upload their data for visualization and analysis. Users need to create a file in a tab-delimited text format (details are available on the website). Upon upload, the data is immediately available for examination. The user is provided a unique URL to access the data, which also makes it simple to share the data with collaborators.

### Custom Integration

The plotting library has been designed to make it easy for developers to integrate iCanPlot into their web applications. It has already been added to eXframe [10], a web repository for genomic data where iCanPlot can be used to easily view data collected from several labs. The source code for the plotting library and other supporting scripts are available on Bitbucket where other developers may contribute to the code to improve it further or fork it and adapt it for their own specific uses. Details of usage and documentation are available online.

## Availability and Future Directions

### Availability, source code and supplementary information

www.icanplot.org

### Requirements

Any browser that supports HTML5 (Firefox 4, Chrome 12, Safari 5, Internet Explorer 9)

### Implementation

JavaScript (using HTML5 Canvas element) and python

### Future directions

We plan to add more types of visualization and analysis modules. We encourage other developers to participate in this effort.
